# Comparison of Digital Self-Assessment Systems and Faculty Feedback for Tooth Preparation in a Preclinical Simulation

**DOI:** 10.3390/ijerph182413218

**Published:** 2021-12-15

**Authors:** Milan Stoilov, Lea Trebess, Markus Klemmer, Helmut Stark, Norbert Enkling, Dominik Kraus

**Affiliations:** Department of Prosthodontics, Preclinical Education and Dental Materials Science, University Hospital Bonn, 53111 Bonn, Germany; lea.trebess@ukbonn.de (L.T.); klemmers@aol.com (M.K.); helmut.stark@uni-bonn.de (H.S.); norbert.enkling@ukbonn.de (N.E.); dominik.kraus@ukbonn.de (D.K.)

**Keywords:** digital assessment, self-assessment, compare software, dental education, digital dentistry

## Abstract

Background: Regarding the new dental licensing regulations in Germany (AOZ), this study evaluated the effectiveness of two different digital tooth preparation validation systems in comparison to traditional faculty feedback. Methods: Participants were randomly divided into groups: Faculty Feedback (FF: n = 33), PrepCheck^®^ (PC: n = 32) and Dental Teacher™ (n = 32). Students had the task to prepare tooth 16 for a retentive full-cast crown. Preparations could be repeated as often as desired. Feedback was provided either by faculty staff or by digital validation systems only. Exams were conducted and graded by two independent and experienced examiners. A survey was performed to evaluate the assessment concepts. Results: No statistical difference in examination performance between groups could be observed. Nevertheless, the survey showed participants preferred consulting the faculty staff rather than the digital validation systems. Students preferred practising with DT rather than with PC. Conclusions: Although both classical and digital methods showed comparable results regarding the preparation examination performance, direct feedback by the faculty staff is still appreciated by the students. A combination of both methods is mandatory since demonstration and advice by the teacher is needed. However, digital tooth preparation validation systems are predestined for free practice sessions, providing self-assessment.

## 1. Introduction

Over the last two decades, digitisation has increased enormously in everyday life. Improved computing power and graphic visualisation have allowed the implementation of various innovations. This development affects many areas of modern healthcare, aiming for higher efficiency, facilitated clinical decision-making and quality assurance. In dentistry, CAD/CAM (computer-aided design/computer-aided manufacturing) technology was first introduced in 1983 by Francois Duret [1]. In 1987, the CEREC^®^ (CEramic REConstruction (Dentsply Sirona^©^, Bensheim, Germany) system was revealed by Werner Mörmann and Marco Brandestini as the first functioning chairside system on the market [2]. Since then, digital dentistry has made a great leap with the widespread use of intraoral scanners, CAD software and various manufacturing techniques. Simultaneously, the demand for digital systems in dental education has evolved [3]. Technology-based teaching includes systems such as computer-assisted learning (CAL) and computer-assisted simulation (CAS). It promises an interactive approach to learning and allows students individual time management due to good availability [4]. To date, two basic approaches of digital dental education systems are available: digital evaluation systems, which provide feedback on the students’ preparation (e.g., PrepCheck^®^ (Dentsply Sirona^©^, Bensheim, Germany)) Dental Teacher™ (KaVo^©^, Biberach, Germany), Compare^©^(Planmeca^©^, Helsinki, Finland)) and force-feedback-based simulators, which use a haptic device and virtual models of human teeth or oral cavity as a platform enabling the practice of dental procedures (e.g., Dente/SIMtoCare^®^ (Vreeland, Netherlands), HapTEL™(Birmingham, England), Forsslund System^©^(Stockholm, Sweden)). Virtual reality simulators should provide the opportunity for integrating clinical case scenarios in the operative teaching environment and facilitate tactile diagnostic skills [5]. Defined tasks should be repeated in a safe environment to improve fine motor skills and hand-eye coordination until a satisfactory level is reached [6,7]. Some studies approve virtual simulators as a supplement in the pre-clinical environment, as students seemed to perform well using a virtual simulator. Nonetheless, these systems cannot yet replace phantom heads [8,9,10].

Therefore, up to now, self-assessment systems such as the PrepCheck^®^ (Dentsply Sirona^©^, Bensheim, Germany), Dental Teacher™ (KaVo^©^, Shinagawa, Tokyo) and Compare (Planmeca^©^, Helsinki, Finland) are predominantly used in dental training. They provide an interface between self-assessment, practising intraoral or extraoral scanning and the use of a real phantom head. Park et al. [11] described them as “useful” in bridging the discrepancy between learning styles and to support students´ self-assessment. Moreover, students would appreciate instant, objective and especially visual feedback on their deficiencies [12,13]. Rosenberg et al. [4] stated computer-assisted training is even superior or at least equivalent to conventional methods. It would reduce the workload for faculty members [14,15] and promise a more objective evaluation [16,17]. Especially with the upcoming introduction of the new licensure requirements for dentists in Germany (AOZ), practice time for students will be drastically reduced. By using digital systems, free practice time could be generated, in which students could perform preparation exercises independently of the course time and the staff supervision.

However, many faculties are still hesitant when it comes to implementing digital technologies in education. They seem to shy away from the high costs and major maintenance effort [18], as permanent adaptation and adjustment to future technologies is mandatory [19]. Since the purchase of these systems depends on their effectiveness [20], the question inevitably arises as to the justification of these systems in dental education.

The aim of this study was, therefore, to investigate the effects of two different digital assessment systems on the preparation skills of inexperienced preclinical dental students at the University of Bonn. Most importantly, a comparison between the traditional faculty feedback and the digital approach has been performed.

## 2. Materials and Methods

The present experimental research study was carried out at the Department for Prosthodontics, Preclinical Education and Dental Materials Science of the University Hospital of Bonn. A total of 97 dental students (51 female and 46 male) participated in the voluntary study. Previously, a power analysis was conducted based on a previous small-scale study (total of 34 participants). The calculation was based on the detection of score differences between the study groups (ANOVA design). Assuming a probability of Type I error of 0.05, a standard deviation of 2.3 grades (estimation based on the score distribution in the previous study) and a minimum detectable effect size of 1,5 grades (dRMSSE = 0.25), to achieve 80% power, a sample size of n = 35 per group was calculated. However, limited access to students resulted in a maximum sample size of n = 33/32 (total of 97 participants) per group. According to the maximum available sample size, while maintaining the values for the other parameters, a minimum detectable effect size of 1.5 grades (dRMSSE = 0.4) was calculated for this study (SPSS Statistics 27, IBM^®^, Armonk, NY, USA). Informed written consent was obtained from all students prior to initiation of the study. An ethics vote was obtained from the ethics committee of the medical faculty of the University of Bonn (number 159/20). Inclusion criteria were defined as follows:

- Students of the 1st–3rd semester;
- Students without preparation experience;
- Students who have successfully completed the technical propaedeutic course;
- Students who have not participated in the fixed prosthodontics preclinical course.

Students had the task to prepare the first maxillary right molar (16) for the reception of a full cast crown, following clinical preparation rules as it often needs early prosthodontic reconstruction. All participants attended an introductory lecture on preparation, ergonomics and operation of the digital evaluation systems and scanners, respectively. In addition, a live demonstration was given on ergonomics and the correct positioning of the phantom head. For randomisation, the participants were assigned numerical values from “1” to “97” and divided into three groups. Group “FF” (n = 33) was conventionally supervised by faculty staff, using the glance and grade (global) method [9,15]. Faculty members had at least two years of professional experience and were therefore familiar with the requirements and the procedure. Group “PC” (n = 32) used the PrepCheck^®^ system V 3.0 (Dentsply Sirona^©^, Bensheim, Germany) and group “DT” (n = 32) the Dental Teacher™ system V 4.0 (KaVo^©^, Biberach, Germany) to get feedback on their preparations. The participants had to scan and evaluate their preparations by the respective digital evaluation system.

**Figure 1:**
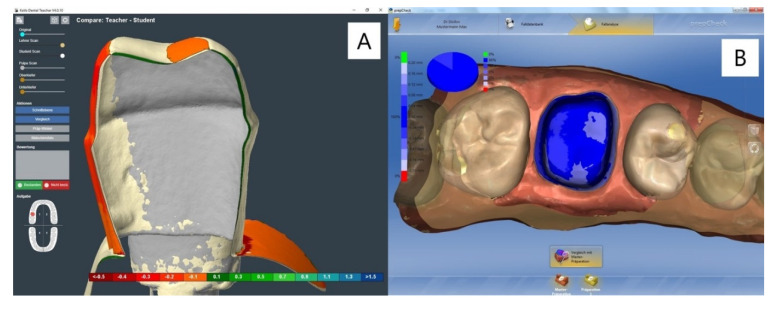
Dental Teacher™ (**A**) showing the cross-section view and PrepCheck^®^ (**B**) showing a proper superimposition of two preparations. Both systems use a colour scale to indicate matching and mismatching areas between the master and student preparations. A perfect match is shown with the PrepCheck^®^ system (**B**). The cross-section view of the Dental Teacher™ system shows an axially and occlusally overextended preparation (**A**).

Both systems are known to be used in pre-clinical dental education. Students scan their prepared training tooth and compare it with a master preparation specified by the teacher. The software superimposes the preparations computing an analysis of the preparation angles, cross-sections, undercuts, metric distances between the preparations, total substance removal, margins, and width of the chamfer. The differences are displayed visually, using a colour scale and by indicating measured angles and metric values (Figure 1). Students can view the models from all directions and use the zoom function, which allows a more precise view. Both systems enable the comparison of all types of preparations. Veneer, inlay, onlay, crown and bridge preparations are the most practised.

The PrepCheck^®^ system (Dentsply Sirona^©^, Bensheim, Germany) is based on the CEREC^®^ system (CEREC^®^ AC and CEREC^®^ InLab software) and represents an extension through the integration of the PrepCheck^®^-app. It uses either the Omnicam^®^ (Dentsply Sirona^©^, Bensheim, Germany) or the Primescan^®^ (Dentsply Sirona^©^, Bensheim, Germany) as the intraoral scanning device. In this process, students must create a patient and follow the exact CEREC^®^ -workflow. This has the advantage that students already simulate clinical practice. In addition, as the system is combined with a cart, it can be placed directly at the students’ workstations. However, the scanning process itself is quite time-consuming, as it requires several steps within the software.

The Dental Teacher™ System (KaVo^©^, Biberach, Germany), on the other hand, represents a stand-alone system that is based on a desktop scanner (ARCTICA^©^ Autoscan (KaVo^©^, Biberach, Germany)). The scanner is connected to a computer by which it is operated. The user interface of the software is simple and intuitive. The scan quality is independent of the students´ skills, and the feeding of the preparation is much faster. Since it is a desktop scanner, the system is not mobile. It requires a fixed location and cannot be used in different rooms.

Before the actual preparation exercise, students were handed out a model of the idealized preparation of tooth 16 (“master preparation”) as visual orientation. They should then perform a preparation according to this template, without adjacent teeth. The preparations were scored by an experienced faculty member using a scale ranging from “0” to “15”, whereby “0” meant “F” and “15” meant “A+”. The initial aim was to evaluate the basic ability of the students to implement shapes in a subtractive procedure before beginning the course. Thus, ensuring a balanced distribution of the participants within each group. A time limit of two hours was given for this first exercise. Beyond that, preparation guidelines for a full-cast crown on tooth 16 were determined as follows: an even substance removal of 0.5 mm circular and 1 mm occlusal. A functional chamfer of approximately 45° and an aesthetic chamfer should be prepared. The preparation margin should be located approximately 0.5 mm supragingivally, and a preparation angle of 4–6° or a convergence angle of 8–12° should be adhered to.

The participants then had the opportunity to practise the preparation of tooth 16 in two free practice sessions. The guidelines, previously taught, should be followed and applied. The aim of this exercise was to be able to perform a safe preparation of tooth 16 for a full-cast crown and thus pass the final preparation exam at the end of the study. During the free practice time, the preparation could be practised and repeated as often as needed. Students were also provided as many teeth as desired. Feedback could be obtained in accordance with group affiliation by the faculty members (group FF) or by the digital evaluation systems (groups PC and DT), at any time and as often as needed.

Models from KaVo^©^ (Upper and lower Basic, Biberach, Germany) and high-speed handpieces (Expertmatic Lux E25 L, KaVo^©^, Biberach, Germany) were provided as part of the study. Diamonds of grit 125 μm (green) and 40 μm (red) with diameters of 010 (1.0 mm) and 012 (1.2 mm) (Horico^®^, Berlin, Germany) were also provided. During the practice, each participant had his own phantom workstation equipped with a suctor, phantom head, rubber mask, air/water syringe, mirror, probe, and tweezers. For documentation reasons, each participant received an identity card, on which group membership, number of used teeth and frequency of contact with the faculty member or scanner were marked. The personal identification number was also noted on the card, which enabled blinded evaluation.

On the last day of the study, a final exam was performed. This involved preparing tooth 16 for a full-cast crown under exam conditions and according to the learned specifications. Each group was given two hours of time. Afterwards, the prepared tooth and the neighbouring teeth were collected and archived in accordance with the code number assigned on the ID card to ensure blinded evaluation.

The preparations of the final exam were evaluated by two chief residents, one in charge of the preclinical and the other of the clinical education, to ensure balanced evaluation. Both individuals had average professional experience of more than ten years. Grading was performed using the same scale as in the preliminary exercise mentioned above (“0” = “F” and “15” = “A+”). The preparations were presented to both examiners independently. No information on the student was given; only the teeth with the corresponding index numbers were presented. Grading was then performed by visual inspection using magnifying dental loupes and a dental and periodontal probe. Evaluation criteria were based on clinical parameters for successful preparation: substance removal, amount of occlusal reduction, damage to the adjacent teeth, finish line quality, preparation angle and surface roughness (Table 1). The decisive parameter that had to be evaluated was whether a casted crown could be fixed to the prepared tooth. If this criterion was not met, grade “D” has been awarded at best.

At the end of the study, participants were handed out questionnaires to provide feedback based on their impressions (Table 2). Each individual item had to be evaluated using the six-point Likert-type scale, where “1” meant strongly agree and “6” full disagreement. Aside from that, the possibility of describing further remarks and impressions using a free text box was given. The evaluation was performed blinded and by an independent staff member. Results were then summarised and presented to the investigator. The statistical analysis of the results was carried out with “Excel^®^” (Microsoft Corporation^©^, Redmond, WA, USA) and the statistics software “Prism 5™” (GraphPad Software^©^, Inc., San Diego CA, USA). The significance level used was *p* < 0.05. Inter-rater agreement was determined by calculating Wilcoxon signed-rank test (Spearman approximation) and Cohen’s weighted kappa (κ).

## 3. Results

**Figure 2:**
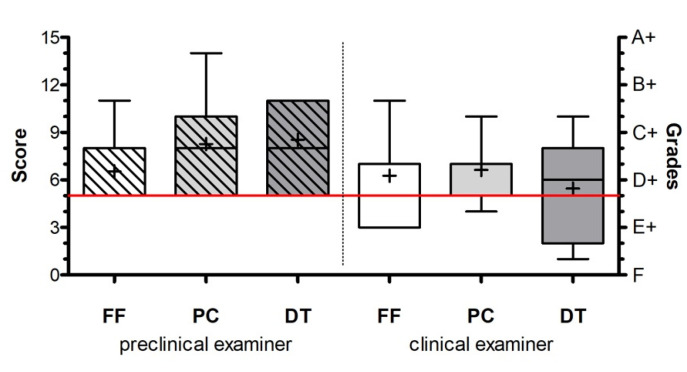
The horizontal red line shows the pass mark of the exam (grade “D” or score 5). Mean ± SEM (n per group = 33(32)) were calculated and one-way ANOVA and the post-hoc Dunn multiple comparison test were applied (*p* < 0.05).

**Figure 3:**
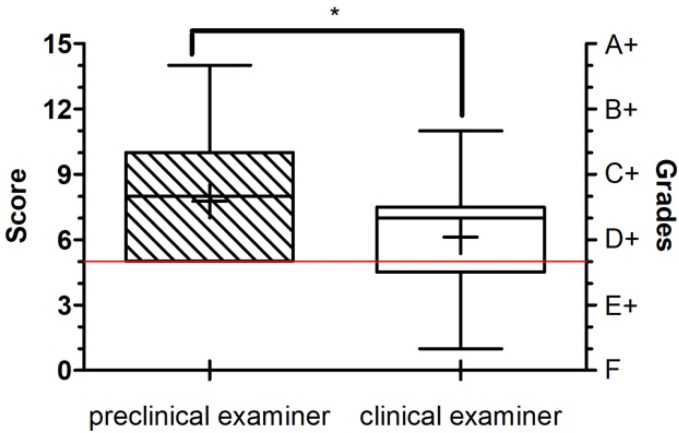
The horizontal red line shows the pass mark of the exam (grade “D” or score 5). Mean ± SEM (n = 33(32)) were calculated and Wilcoxon signed-rank test (* = *p* < 0.05) and Spearman’s rank correlation (*p* = 0.4057) were performed. “*” demonstrates the significant difference between the two examiners, with the preclinical examiner giving higher grades.

All 97 participants completed the exercises, the final exam, and the evaluation form. No drop-outs were documented. Table 3 present the scores evaluated by the two examiners and the score distribution within each group (mean, SD, median). Interestingly, the preclinical examiners evaluation revealed higher ratings in the PC (8.188 vs. 6.625) and DT (8.469 vs. 5.500) groups than in the FF (7.091 vs. 6.273) group. However, statistical analysis did not expose the difference in the performance within groups (Kruskal–Wallis and Dunn´s multiple comparison test; *p* < 0.05) (Figure 2). Comparison of both examiners showed significant differences in their ratings, with the preclinical investigator giving higher grades on average (Figure 3). Assuming grade “D” as a minimum requirement for passing the exam, the preclinical examiner did not rate below this grade, whereby 23 participants (22%) failed the exam according to the clinical examiner´s rating. In accordance with that, Cohen’s weighted kappa showed only a slight agreement between the examiners (weighted κ = 0.268).

**Figure 4:**
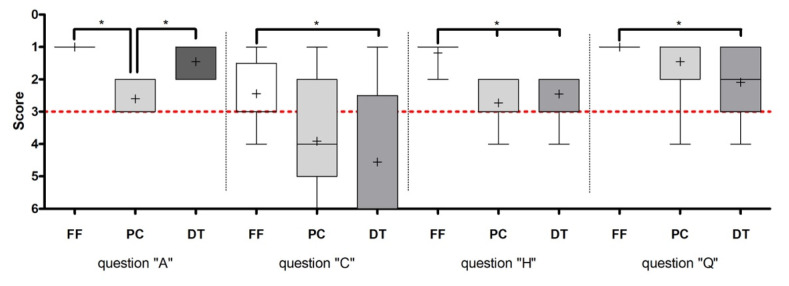
The horizontal red dash line visualises a “slightly agreement” (3 points) according to the Likert-type scale. Mean ± SEM (n per group = 11) were calculated and Kruskal–Wallis test (* = *p* < 0.05) and Dunn multiple comparison test (* = 0.05) were performed. “*” indicates significant statistical differences between the groups.

Analysis of the questionnaires revealed statistically significant differences for questions A, C, H and Q. Table 4 and Table 5 present the percentage distribution for each group and question. To compare the scores, Kruskal–Wallis and Dunn´s multiple comparison tests were performed and presented as box plots (Figure 4). All participants of the faculty feedback group (100%, n = 33: “full agreement”) rated working with the faculty staff as uncomplicated (*p* < 0.001). The comparison between the computer-assisted groups showed that students preferred practising with the Dental Teacher™ System than with the PrepCheck^®^ System (question A) (*p* < 0.001). The rating of the respective methods was significantly in favour of the faculty feedback (question H) (*p* < 0.0001). Almost all participants of the faculty feedback group (FF) rated this method as “very good” (82%), whereas participants of the other groups rated their methods just as “good” (Dental Teacher™, 63%) and as “satisfactory” (PrepCheck^®^, 53%).

Scheme 94. Those in the PrepCheck^®^ (72%) and Dental Teacher^™^ (68%) groups had a similar opinion (question Q) (*p* < 0.0262). Question “C” reveals that participants tend to seek advice from the faculty members (87%) rather than use the Dental Teacher^™^ System (63%) for the evaluation of their preparations (*p* < 0.0422).

## 4. Discussion

The present study investigated the effectiveness of the “PrepCheck^®^” and “Dental Teacher™” systems in comparison with traditional faculty feedback. The evaluation of the results proved no statistical difference for the scores achieved.

The type of evaluation had no influence on the performance of the participants. Furthermore, digital preparation assistant systems appear to be equally effective as traditional glance and grade methods [4,21]. Wolgin et al. [22] and Sadid-Zadeh et al. [23] claim that immediate feedback via virtual assessment software may be as effective as faculty feedback. Learning systems could thus have a beneficial effect on preclinical training, to promote independent learning strategies and to improve the self-assessment of students. In addition, they could ease high student–teacher ratios and reduce the faculty workload [14,15]. Another Study by Nagy et al. [13] shows a significant increase in the learning curve for complex cavity preparations. The Dental Teacher™ software is described as a tool to deepen the understanding of preparation shapes and dimensions.

However, Gratton et al. [24] criticise the moderate correlation of the evaluation by the faculty members and the digital systems. Digital systems compute deviations from the specified preparation that have no clinical relevance. An experienced dentist could recognise this circumstance and provide more appropriate feedback. It is further argued that the faculty feedback appears to be faster and more valuable to the students, as the final evaluation is performed by the faculty anyway. Moreover, the effectiveness of the systems varied depending on the individual skills of the user, the user’s experience with the system and the technical support. According to Hubbard et al. [25] and Cho et al. [26], low-performing students seem to be unable to recognise their weaknesses or poor performance.

In summary, it is rather suggested to synergistically use the positive effects of both approaches. Digital systems have the great advantage of providing objective and non-influenceable feedback. Beginners, in particular, are able to self-assess their work independently and repeat initial preparations, still showing major deficiencies. At this stage, errors appear to be obvious and do not require any intervention by the faculty. Using digital assessment, students should be able to recognise and improve their deficiencies based on graphic visualisation. In the sense of a pre-course or during free practice, preparation skills could be improved so that the regular course time could be used more effectively, and the initial frustration of the faculty members would be reduced. In addition, free practice would be beneficial for an upcoming exam.

However, this principle can only be successful if students are self-directed in learning. In line with Knowles [27], self-directed learning is a process in which students take the initiative to diagnose their learning needs, formulate learning goals, identify human and material resources for learning, select and implement appropriate learning strategies and evaluate learning outcomes.

This is important because it helps students develop the capacity for lifelong learning, an essential skill expected of a health professional [28]. Self-directed learning should also occur in formal contexts, such as college courses. Teachers should then promote self-directed learning by designing creative assignments that challenge learners to be more independent and make decisions about their learning. The role of faculty has changed from that of an impeccable teacher to that of a guide. This role change is one of the most important factors in the success of self-directed learning [28]. Thus, during free practice time, the supportive role of an assistant dentist would be required, rather than that of an examiner, to motivate students to engage in self-directed learning.

Whilst self-directed learning can be beneficial to the student, the presence and input of a clinical teacher promote the social learning theory of the community of practice. Lave and Wenger [29] defined it as a group of people who share a concern or passion for something they do and learn how to do it better through regular interaction. Dental students and faculty should therefore be part of a group, e.g., within a practice course. This group has the deliberate goal of gaining knowledge in a specific area (e.g., preparations). By sharing information and experiences within the group, members learn from each other and can develop personally and professionally. Furthermore, best practices are exchanged, information is reused and members learn from previous mistakes [29].

Considering the amendment of the dental licensing regulations in Germany (AOZ) in 2021, a massive reduction of the curricular time is expected. However, the preclinical education of future dentists will be affected by this, which is unfortunate since this is where fundamental knowledge and skills are taught. As the primary goal still remains to ensure the quality of education and dental care, the student–faculty ratio is to be improved from 20:1 to 15:1. The effectiveness of this measure remains to be proven. It is certain that an improved ratio cannot be implemented at the expense of the number of study places. It seems sensible to introduce digital evaluation and practice systems that are independent of supervision [17]. In the sense of a freely accessible simulation laboratory, they could allow more practice time without having to reduce the number of study places or even increase the number of faculty staff. Students could thus learn autodidactically and evaluate their own performance self-critically [11,30].

Nevertheless, the major disadvantage of digital evaluation is the lack of practical support and face-to-face formal feedback. Only experienced faculty staff can provide direct constructive and meaningful feedback to students on implementing correct workplace ergonomics, handling of dental equipment to include the fast and slow handpieces, manual dexterity and hand-eye coordination skills, finger rest and tactile drilling. Live demonstrations and explanations of treatment steps or practical procedures are carried out right at the workplace. Most importantly, students are taught to comprehend how relevant errors occur [31]. To date, such didactic challenges can be performed exclusively by trained dentists.

It is common for dentists to be involved in the teaching and mentoring of dental students. Typically, students learn how to perform a dental procedure by first observing and then practising it through a hands-on approach. Despite this, learners may still be unfamiliar with a particular technique or have difficulties performing certain tasks. Teachers should then provide the necessary assistance or take control to perform the task adequately and guide students in the right direction [32]. Digital assessment systems are not able to perform this task since they measure only absolute values and cannot provide “feedback in action” [33]. However, these real-time comments from the teacher are very important to allow students to reflect on the impact of their actions, which is referred to as “reflection in action” [33]. Feedback should therefore be given as close as possible to the learner’s performance in order to have the greatest impact [34].

Moreover, a self-directed approach to learning using digital supervision by automated assessment systems is impersonal and distant, as they focus solely on the practical performance of the students (hard skills). Students are expected to not only be clinically competent upon graduation but also to exhibit good soft skills in order to serve society at large [35]. Clinical, scientific and interpersonal skills have been recognised as important components of the dental curriculum [35]. Among others, communication skills [36,37,38,39,40], critical thinking [41,42], teamwork [43], leadership [44], professionalism [40,45], life-long learning [46] and entrepreneurship [47] have been described in the literature as soft skills. They cannot be taught or evaluated by a digital self-assessment tool, as personal interaction with the faculty staff is needed.

Overall, a synergistic workflow between faculty feedback and digital evaluation is recommended. Faculties should combine the advantages of each method to provide objective and effective feedback.

Since the onset of the SARS CoV-2 pandemic, dental students´ learning opportunities have been severely restricted. Universities have had to adapt and rely on digital technologies and independent, self-directed learning approaches to maintain necessary educational requirements. Thus, several educational programs have had to undergo a sudden transition from face-to-face instruction to virtual or E-learning, which has presented some challenges in achieving the intended learning outcomes [48]. In dental education, disproportionate disruption occurred, especially in learning fine motor skills for clinical training [49]. Dental institutions adapted to the situation by integrating videoconferencing options for training. The use of online platforms, video demonstrations, live or streamed videos and recorded lectures are some of the virtual teaching methods that dental schools have adapted [50].

Currently, teaching manual skills cannot be easily implemented digitally or virtually. Hands-on courses must continue to be offered by providing didactic online instruction and practising manual dexterity and fine motor skills in small groups. In this context, the use of the systems presented in this study only avoid contact with the teachers. Students would still meet in the course room. Online platforms would allow for theoretical training, but their use in practical training remains questionable. Some dental disciplines appear to work well with online instruction, while others require students to complete preclinical hands-on training prior to actual clinical work to ensure competency in managing patients with dental disease [51]. Given advances in technology, simulation of clinical environments through modalities such as haptics, force feedback and virtual reality-based simulation has become possible without actually being in a clinical setting.

However, these systems are not widely available yet and require further development before they could replace traditional training methods. Considering the massive impact of this pandemic on the education of future dentists and society, investment in such technologies shall be strongly recommended.

The assessment of students’ practical skills has been a subject of controversial debate in dental education in the past. The lack of objectivity and inconsistency of the examiners have always been criticised [22,52,53]. Considering this, students tend to choose those faculty members who are known to give a better scoring, which could result in a potential disadvantage for those who do not selectively choose their examiner. The assessment is then perceived as arbitrary and is thus affected. Moreover, a negative impact on students’ confidence and performance is observed [54]. The well-known “glance and grade” method (visual assessment and verbal feedback) is still widely used and means of choice in assessing dental students’ practical performance [2,55,56]. In fact, however, consistent assessment is instrumental in the successful teaching of practical skills, theoretical knowledge and motivated learning [57,58].

The present study analysed the inter-rater reliability of two examiners depending on their functional area (preclinical and clinical education), with the result of a significantly better scoring by the preclinical examiner and just a slight agreement of the examiners.

This observation seems understandable since the preclinical examiner deals with less experienced students. A process of habituation seems to have occurred, as a result of which poor preparations are evaluated with higher scores. Often, only the minimum requirements are met, yet the grade “good” is awarded. Students from the clinical study section, on the contrary, have already passed all practical courses and exams in order to work on patients. That is why a higher level of quality is expected and demanded by the clinical examiner. He is accustomed to more experienced and more capable students.

In order to enable a fundamentally better agreement within examiners, some authors have already recommended the use of standardised evaluation forms with the same criteria for all examiners. Regular calibration of faculty members by conducting joint training according to these criteria should be mandatory [59,60]. The introduction of digital technologies could facilitate the calibration of the faculty staff and chief examiners, providing objectivity in the notoriously subjective process of evaluation. They are widely accepted among students as they offer more focused and objective evaluations than their instructors [15,61].

Lastly, a recent study by Miyazono et al. [62] shows that purely examiner-based evaluation was associated with significantly higher inter-rater disagreement. A complementary use of evaluation software would increase objectivity and reliability.

The question of whether practising with the computer assistant or faculty members was easy (Table 3; question A) has been rated positively overall. Nevertheless, graduations can be seen. Faculty feedback received the best rating. All participants fully agreed that working with the faculty staff was easy. This positive perception might be derived from the fact that they have become familiar with the interaction with the faculty staff during the former propaedeutic course. Participants in the PrepCheck^®^ and Dental Teacher™ groups were confronted with an entirely new situation that differed from the usual procedure and had to adjust to an entirely new concept. Beyond of that, they were left alone with the decision when to start over with a new tooth or when to perform a new scan. Therefore, results were not as good as in the faculty feedback group. Only 55% of the Dental Teacher™ group agreed fully, and just 40% of the PrepCheck^®^ group partially agreed when asked whether working with the system was easy. The scanning process with the desktop scanner-based Dental Teacher™ system was found to be easier. The login and scanning process of the PrepCheck^®^ system appeared complex, as the workflow is strongly based on the CEREC^®^ system. Significantly, more steps are necessary to obtain the comparison of the preparations, which is time-consuming. Nevertheless, this system allows training digital impressions with an intraoral scanner.

The Dental Teacher™ software, in contrast, is based on a simple acquisition software combined with a straightforward compare-software. The user just needs to place the prepared tooth into a scan base and start the scanning process after logging in. However, intraoral scanning cannot be practised.

Regarding the question of whether participants had to rescan or visit the faculty staff several times to complete a preparation (Table 4; question C), participants in the faculty feedback group showed intermediate steps to get fast feedback. Again, the situation was different for the groups using the digital evaluation systems. In both groups, participants indicated that they rarely rescanned their preparations. The reason for this could be the generated feedback, which initially appears to be sufficient to avoid serious mistakes. Another reason might be the irregular availability of the devices since only one PrepCheck^®^ and Dental Teacher™ unit were available. Therefore, participants had to cope with waiting time. This circumstance was intensified by the lack of experience in using the systems. In contrast, the waiting time was significantly shorter as the experienced assistant dentist could provide fast feedback. Due to the small number of subjects, feedback could be obtained easily and quickly.

It should be considered that the inclusion criteria for participation in this study excluded any preparation experience. Consequently, it seems understandable that students contacted the faculty staff more often. Besides the evaluation of the preparation, instructions were also given to improve the quality of the preparations. This included correct finger or hand rest, handling of the high-speed handpiece, positioning of the phantom head and, most importantly, a live demonstration. This was felt to be necessary by all participants (Table 4: question Q). The compare software is not capable of doing this. It can only show errors and deviations compared to the master preparation. The software cannot yet generate helpful hints for correction or improvement.

Finally, the participants were asked to rate the used support concept with a scale from “very good” to “very poor” (Table 4: question H). In total, the concepts received an overall satisfactory rating. However, the assistant dentists received the grade “very good”, whereas the digital systems received the grades “good” (Dental Teacher™) or “satisfactory” (PrepCheck^®^). This result supports the argumentation above and confirms the overall impression gained.

## 5. Conclusions

Under the limitation of this study, it may be concluded that the sole use of digital evaluation systems for the preclinical education of dentistry students seems to be initially as effective as the traditional faculty feedback. The achieved scores of the exam did not differ significantly within groups. However, during the free practice time, it could have been observed that students using the digital assessment tools had major problems recognising and optimising the deficiencies of their preparations. This was particularly the case during the early stages of education; students would therefore need demonstration and proper advice by the teacher to learn and to identify deficiencies. A combination of traditional and objective digital feedback is therefore mandatory. However, digital systems should be used to enable independent practice outside of regular courses and thus prolong practice time without needing an extra workforce. This would benefit independent learning and reduce faculty workload. Furthermore, calibration of the faculty staff and the examiners could be performed using the compare software, whereby the overall agreement could be increased. A standardised evaluation form with valid criteria should be used and discussed. Since the scanning process was time-consuming, valuable practice time has been lost. Therefore, a less complex workflow is required, and a sufficient number of units should be purchased (at least 1:5) since students had to cope with waiting time.

In summary, the array of modern digital equipment in dentistry has increased significantly. Full digital workflows and innovative dental materials offer advantages such as optimal mechanical resistance, excellent aesthetic and optical properties and reliable accuracy and precision, widening the clinical scenario and allowing for innovative and less invasive restorative solutions.

Hence, future dentists expect an updated education utilising modern dental technologies. Therefore, educational institutions urgently need to embrace digital technological innovations to help enhance the overall quality of dental education and to keep up with the overall development. However, the dynamic development in digital dental technologies could be affected by rapid obsolescence and replaced by even more innovative systems. Institutions will have to deal with constant updates to take advantage of the potential of these systems. In this context, further research on this topic is needed. Future studies with larger study populations and a larger number of examiners from both preclinical and clinical settings are recommended.

## Figures and Tables

**Table 1:** Criteria assessed for evaluation of teeth prepared during the exam. Examiners had the option to rate with “+” (yes), “−“ (no) or “0” (neutral). A total grade from 0–15 points resp. F–A+ could be awarded.

Assessed Criterion	Score	
Is a crown attachable?	+/−	
Substance removal?	Circular	Occlusal
	+/− or “0”	+/−or “0”
Damage to adjacent teeth?	+/− or “0”	+/− or “0”
Finish line quality?	+/− or “0”	+/− or “0”
Preparation angle? (Undercuts? Conicity?)	+/− or “0”	+/− or “0”
Surface roughness?	+/− or “0”	+/− or “0”
**Grade Scale**	**Total Grade**	
0 to 15 | F to A+		

**Table 2:** Questions handed out to the students of each group.

1 = Full Agreement 6 = Full Disagreement	(1)	(2)	(3)	(4)	(5)	(6)
A. Practising with the computer-assistant or faculty members was easy?						
B. Working with a computer appears to be difficult?						
C. To complete the preparation, you have scanned several times/presented your preparations to the faculty members and made corrections?						
D. Faculty members cannot be replaced by a computer assistant when evaluating the preparations?						
E. I would like practise with a computer assistant during the Phantom course?						
F. A computer assistant is helpful when practising for your practical exam?						
G. A computer assistant should be used by the faculty members to evaluate the preparations in the Phantom course?						
H. Please grade the method or system you have worked with!						
I. Self-evaluation during preparation is particularly important?						
J. Final evaluation of the quality of the preparation is particularly important?						
K. It is important to assess why your performance has led to the achieved result?						
L. For improvement of your preparation skills, you will use your written records, books, and digital content?						
M. For improvement of your preparation skills, you use three-dimensional models?						
N. For improvement of your preparation skills, you look at pictures of prepared teeth?						
O. For improvement of your preparation skills, you watch videos of how a tooth hast to be prepared?						
P. For improving your preparation skills, it is important for you to look at the preparations of your colleagues?						
Q. For improving your preparation skills, it is important to watch a faculty member prepare a tooth?						
**Free text box**						

**Table 3:** Assessed scores by the two examiners divided into preclinical and clinical examiners and into groups (FF, PC, DT). Each examiner rated 97 preparations. The mean, SD and median were calculated, and Cohen’s kappa (κ = 0.122), Cohen´s weighted Kappa (κ = 0.268) and Spearman’s rank correlation (ρ (r_s_) = 0.4057) were performed. “***” demonstrates the significant difference between the two examiners, with the preclinical examiner awarding higher grades.

**Scores of the Examiners (Final Exam)**																									
		preclinical examiner (n = 97)												clinical examiner (n = 97)										ρ (r_s_)	
mean		7.907 (“C”)												6.134 (“D+”)										0.4057 ***	
SD		± 2.566												± 2.486											
median		8.000 (“C”)												7.000 (“C-“)											
**Cohen´s Kappa**																									
κ = 0.122						SE = 0.039							95%-CI = 0.045 to 0.198						weighted κ = 0.268						
**Examiners´ Score Distribution (Final Exam)**																									
A+	A		A-	B+			B		B-	C+		C		C-	D+		D	D-		E+		E	E-		F
15	14		13	12			11		10	9		8		7	6		5	4		3		2	1		0
preclinical examiner																									
	3						22		6			27		6			33								
clinical examiner																									
							3		9	3		9		26	9		15	5		9		6	3		
**Groups´ Score Distribution (Final Exam)**																									
preclinical examiner														clinical examiner											
		FF (n = 33)			PC (n = 32)			DT (n = 32)			*p*-value			FF (n = 33)		PC (n = 32)					DT (n = 32)			*p*-value	
mean		7.091			8.188			8.469			0.0652			6.273		6.625					5.500			0.3967	
SD		±2.018			±2.832			±2.622						±2.601		±1.773					±2.885				
median		7.000			8.000			8.000						7.000		7.000					6.000				

**Table 4:** Student responses to questions A to H in accordance with their group affiliation (FF = faculty feedback, PC = PrepCheck^®^, DT = Dental Teacher™). Questions were rated according to the Likert-type scale (1−6 points). Results are presented in percentages.

Question	Group	Score					
		1	2	3	4	5	6
“A” Practising with the computer-assistant of faculty member was easy?	FF	100%					
	PC		40%	60%			
	DT	63%	37%				
“B” Working with a computer appears to be difficult”?	FF			27%	9%	27%	18%
	PC		9%	18%	9%	27%	36%
	DT	9%	18%			27%	27%
“C” To complete the preparation, you have scanned several times/presented your preparations to the faculty members and made corrections?	FF	20%	20%	47%	10%	3%	
	PC	20%	13%	10%	27%	10%	
	DT	10%	10%	17%	17%	13%	33%
“D” Faculty members cannot be replaced by a computer assistant when evaluating the preparations?	FF	36%	27%	18%	18%		
	PC	55%	18%	9%	9%		9%
	DT	55%	18%	18%			
“E” You would like to practise with a computer assistant during the upcoming phantom course?	FF	37%	17%	46%			
	PC	36%	18%	27%	9%		
	DT	36%	18%	27%	9%		9%
“F” A computer assistant is helpful when practising for your practical exam?	FF	36%	27%	27%			
	PC	55%	27%		9%		9%
	DT	45%	27%	27%			
“G” A computer assistant should be used by the faculty staff to evaluate the preparations in the phantom course?	FF		9%	45%	9%	9%	9%
	PC	9%	18%	9%	27%	36%	
	DT	9%	9%	9%	27%	18%	27%
“H” Please grade the method or system you have worked with!	FF	82%	15%				
	PC		38%	53%			
	DT		63%	28%	6%		

**Table 5:** Student responses to questions I to Q in accordance with their group affiliation (FF = faculty feedback, PC = PrepCheck^®^, DT = Dental Teacher™). Questions were rated according to the Likert-type scale (1−6 points). Results are presented in percentages.

Question	Group	Score					
		1	2	3	4	5	6
“I” Self-evaluation during preparation is particularly important?	FF	27%	64%	9%			
	PC	55%	27%	9%	9%		
	DT	27%	55%	18%			
“J” Final evaluation of the quality of the preparation is particularly important?	FF	36%	45%	9%			
	PC	55%	18%	18%	9%		
	DT	18%	27%	36%	18%		
“K” It is important to assess why your performance has led to the achieved result?	FF	100%					
	PC	72%	27%				
	DT	72%	27%				
“L” For improvement of your preparation skills, you will use your written records, books and digital content?	FF	18%	27%	36%	9%		9%
	PC	27%	18%	27%	18%	9%	
	DT	45%	27%	18%			
“M” For improvement of your preparation skills, you use three-dimensional models?	FF	36%	18%	18%	9%		18%
	PC	36%	36%	9%			9%
	DT	18%	45%	27%	9%		
“N” For improvement of your preparation skills, you look at pictures of prepared teeth?	FF	64%	27%			9%	
	PC	45%	36%	9%		9%	
	DT	45%	18%	27%	9%		
“O” For improvement of your preparation skills, you watch videos of how a tooth hast to be prepared?	FF	65%	10%			9%	16%
	PC	72%	18%				9%
	DT	45%	45%	9%			
“P” For improving your preparation skills, it is important for you to look at the preparations of your colleagues?	FF	37%	17%	46%			
	PC	71%	19%				
	DT	28%	44%	18%	10%		
“Q” For improving your preparation skills, it is important to watch a faculty member prepare a tooth?	FF	94%	6%				
	PC	72%	19%	3%	6%		
	DT	68%	26%		6%		

## Data Availability

Not applicable.
